# Metabolic syndrome and cardiovascular disease after haematopoietic cell transplantation (HCT) in adults: an EBMT cross-sectional non-interventional study

**DOI:** 10.1038/s41409-021-01414-7

**Published:** 2021-07-17

**Authors:** D. M. Greenfield, N. Salooja, C. Peczynski, S. van der Werf, H. Schoemans, K. Hill, A. Cortelezzi, M. Lupo-Stangellini, Z. N. Özkurt, M. Arat, B. Metzner, P. Turlure, A. Rovo, G. Socié, M. Mohty, A. Nagler, N. Kröger, P. Dreger, M. Labopin, T. S. Han, A. Tichelli, R. Duarte, G. Basak, J. A. Snowden

**Affiliations:** 1 Sheffield Teaching Hospitals NHS Foundation Trust, Sheffield, UK; 2 Imperial College, London, UK; 3 EBMT Paris Study Office, Paris, France; 4 EBMT Data Office Leiden, Leiden, The Netherlands; 5 Department of Hematology, University Hospitals Leuven and KU Leuven, Leuven, Belgium; 6 University Hospital Southampton NHS Foundation Trust, Southampton, UK; 7 Fondazione IRCCS Ca’ Granda Ospedale Maggiore Policlinico, Milano, Italy; 8 San Raffaele Scientific Institute IRCCS, Milano, Italy; 9 Gazi University Hospital, Ankara/Turkey, Ankara, Turkey; 10 Sisli Florence Nightingale Hospital, Istanbul, Turkey; 11 Klinikum Oldenburg, Oldenburg, Germany; 12 CHRU Limoges, Limoges, France; 13 University Hospital Bern, Bern, Switzerland; 14 Hospital St. Louis, Paris, France; 15 Saint-Antoine Hospital, Sorbonne University, INSERM UMRs 938, Paris, France; 16 Chaim Sheba Medical Center, Tel-Hashomer, Israel; 17 University Hospital Eppendorf, Hamburg, Germany; 18 University of Heidelberg, Heidelberg, Germany; 19 Institute of Cardiovascular Research, Royal Holloway, University of London, Egham, UK; 20 University Hospital, Basel, Switzerland; 21 Hospital Universitario Puerta de Hierro, Madrid, Spain; 22 The Medical University of Warsaw, Warsaw, Poland

**Keywords:** Diseases, Medical research

## Abstract

Metabolic syndrome (MetS) is associated with cardiovascular disease in the general population and is also a potential cardiovascular risk factor in survivors of haematopoietic cell transplantation (HCT). We report an EBMT cross-sectional, multi-centre, non-interventional study of 453 adult HCT patients surviving a minimum of 2 years post-transplant attending routine follow-up HCT and/or late effects clinics in 9 centres. The overall prevalence of MetS was 37.5% rising to 53% in patients >50 years of age at follow-up. There were no differences in rates of MetS between autologous and allogeneic HCT survivors, nor any association with graft-versus-host disease (GvHD) or current immunosuppressant therapy. Notably, there was a significantly higher occurrence of cardiovascular events (CVE, defined as cerebrovascular accident, coronary heart disease or peripheral vascular disease) in those with MetS than in those without MetS (26.7% versus 9%, *p* < 0.001, OR 3.69, 95% CI 2.09–6.54, *p* < 0.001), and, as expected, MetS and CVE were age-related. Unexpectedly, CVE were associated with occurrence of second malignancy. Screening for and management of MetS should be integrated within routine HCT long-term follow-up care for both allogeneic and autologous HCT survivors. Further research is warranted, including randomised controlled trials of interventional strategies and mechanistic studies of cardiovascular risk in HCT survivors.

## Introduction

Metabolic syndrome (MetS) is typically defined as a clustering of five factors including (1) hyperglycaemia (2) hypertriglyceridaemia; (3) low high-density lipoprotein (HDL) cholesterol; (4) hypertension; (5) obesity (measured by high waist circumference) [1, 2]. The prevalence is estimated to be about one quarter of the world population [3]. This cluster of interrelated risk factors has been shown to increase the risk of cardiovascular (CV) disease, diabetes mellitus (DM) and all-cause mortality [2, 4]. Various definitions of MetS have been proposed but currently, an international harmonised definition given by the International Diabetes Federation (IDF) [1] defines MetS as 3 out of 5 risk factors, as follows: abdominal obesity, measured by waist circumference (using population and country-specific definitions): triglycerides (TG) ≥1.7 mmol/l, or drug treatment for elevated levels; high-density lipid–cholesterol with gender specific cut-offs (men <1.0 mmol/l, women <1.3 mmol/l or drug treatment for reduced levels); blood pressure ≥130/≥85 mmHg or drug treatment for hypertension; fasting glucose ≥5.6 mmol/L or drug treatment for DM. Another commonly used definition is the NCEP ATP III definition [4]. For this, MetS is present if three or more of the following five criteria are met (using imperial measures): waist circumference over 40 inches (men) or 35 inches (women), blood pressure over 130/85 mmHg, fasting TG level over 150 mg/dl, fasting HDL cholesterol level less than 40 mg/dl (men) or 50 mg/dl (women) and fasting blood sugar over 100 mg/dl.

One significant practical issue is the variable definition of MetS used across published studies and in clinical practice, generally and in relation to haematopoietic cell transplantation (HCT). Using a population-based study of randomly selected participants, Mancia et al. [5] have previously compared the predictive value of the various definitions of MetS and found this was significantly greater when using the criteria of the American Heart Association [6] and the IDF [1] definitions compared with the that of the National Cholesterol Education Programme Adult Treatment Panel III (NCEP ATPIII) [4] definition. In their literature review, O’Neill and O’Driscoll [7] tabulated the rates of MetS by the two definitions (NCEP ATPIII and IDF) with consistently higher rates using the IDF definition across a number of studies. The IDF method is now proposed to be superior at diagnosing MetS compared with that of the NCEP ATPIII [7].

Compared with the general population, the prevalence of MetS is increased in long-term cancer survivors in both adult [5–8] and paediatric survivors [9–11]. Furthermore, there is evidence of increased morbidity and mortality from CV disease in populations of general cancer patients [12, 13]. Some studies have included HCT recipients, but they have been mixed in their patient populations, variable in their definition of MetS and other endpoints, and in their conclusions. With respect to an increased risk of CV disease after HCT, retrospective studies from the European Society for Blood and Marrow Transplantation (EBMT) found 3.6% of long-term survivors of allogeneic HCT had a cardiovascular event (CVE) in at least one arterial territory; the cumulative incidence of a first CVE 15 years after HCT was 6% which increased to 7.5% in allograft survivors compared with 2.3% post autologous HCT [12, 13]. Another large retrospective study in a North American population reported 10-year cumulative incidences of ischaemic heart disease, cardiomyopathy, stroke and all-cause CV death of 3.8%, 6%, 3.5% and 3.7% respectively [14], with a similar prevalence of CV disease in autologous and allogeneic HCT.

A number of individual mechanistic factors potentially contribute to the increased risk of CV disease following HCT including pre-existing risk factors, pre-transplant treatment, the transplant itself (including graft-versus-host disease, GvHD) and post-transplant treatment and the contribution of immune dysfunction and endocrinopathies [14–25].

Although the precise relationship between HCT, MetS and CV disease needs further clarification, screening for MetS following HCT has been incorporated into international guidelines for long-term follow up of HCT patients [26–28]. Even so, the incidence and impact of MetS as a risk factor for CV disease following various types and intensities of HCT remains unclear.

We therefore conducted a large cross-sectional, multi-centric service evaluation of HCT survivors in EBMT centres working in accordance with international screening guidelines for long-term follow up of HCT survivors [26]. The primary objective was to establish the prevalence of MetS following various types of HCT in consecutive patients returning for routine follow-up over a 1-year period at participating EBMT centres. Secondary objectives were to evaluate the association of MetS with a range of patient and HCT-related variables and discern the discriminatory ability of both MetS definitions (IDF and ATPIII) in the HCT survivor population.

## Subjects and methods

This was a multi-centre, cross-sectional, non-interventional observational study. The study was approved and conducted according to EBMT procedures with data collection and further evaluation coordinated centrally by the EBMT Leiden Data Office, The Netherlands, with statistical analysis performed by the EBMT Office Paris, France. All patients provided written consent at transplant for the collection, transmission and analysis of anonymised data within EBMT.

### Inclusion criteria

Patients transplanted as adults (aged ≥18 years) were eligible for inclusion if they had received allogeneic (myeloablative or reduced intensity) or autologous HCT with curative intent. Patients had to be a minimum of 2 years post HCT and be attending a routine follow-up appointment at one of nine participating EBMT centres. The patients treated with non-curative intent (e.g. patients with myeloma or low grade non-Hodgkin lymphoma (NHL) treated with autologous HCT) were not eligible.

### Data collection

Transplantation-related data for each patient was extracted from the EBMT registry and included age, gender, ethnicity and details regarding allogeneic or autologous HCT with dates, type of conditioning regimen: myeloablative +/− total body irradiation (TBI)/reduced intensity conditioning (RIC), acute GvHD (aGvHD) grade, chronic GvHD (cGvHD) grade, Eastern Cooperative Oncology Group (ECOG) performance status, and details of any second or newly occurring malignancy (including skin cancers) with date of diagnosis. Histological confirmation was not routinely collected.

In advance of the study, the EBMT Leiden Data Office emailed all EBMT transplant centres seeking interested centres to participate. Nine centres agreed to participate in the study and went on to enter patients into the study. No information was gathered regarding the socioeconomic diversity or follow-up schedule of the participating centres. Participating centres also completed study-specific EBMT ‘MED C’ forms for each patient. MED C routine clinical recording included parameters for MetS (such as diabetes, hypertension, dyslipidaemia, waist circumference). In addition, the following were all collected; performance status (ECOG); height (accepting baseline/previously measured height) and weight; evidence of previous CVE based on the categories as defined by Tichelli et al. [29]; family history of premature CV disease (parents <60 years), existing diagnosis of Type 2 DM pre-HCT, menstrual history in women, previous and current smoking and calculation of pack-years smoked (number of packs of cigarettes smoked per day multiplied by the number of years the person has smoked), previous and current alcohol use (using UK guidelines to calculate the number of units per week). Previous or current drug history was also recorded on the MED C form including use of: immuno-suppressive therapy, particularly use of cyclosporine and tacrolimus, sirolimus; corticosteroid dosage including previous or current prednisolone equivalent doses; anti-lipid drugs; anti-hypertensives (including ACE inhibitors, beta-blockers, diuretics used for hypertension not fluid balance); diabetic medications (including insulin and metformin); hormone replacements (including contraceptive use in women). Data were coded under unique patient numbers and all centres submitted completed study data for integration within the EBMT Registry to a central data office (the EBMT Leiden Data Office) by established reporting routes. Definitions of the ATPIII and IDF criteria were as published [1, 4, 5].

### Routine clinical tests

Routine clinical tests. including TGs, HDL and LDL cholesterol, plasma glucose, total testosterone or oestradiol and gonadotrophins, sex hormone binding globulin, thyroid-stimulating hormone and T4, were performed for each patient at the follow-up appointment and results recorded on the MED C form as within range, elevated or lower than local reference ranges. Patients were reviewed in routine follow-up clinics and blood taken under normal conditions according to local clinic arrangements. Fasting blood results were not always available in which case non-fasting plasma glucose cut-offs were used according to the American Diabetes Association guidance [30].

### Statistical analysis

Statistical analyses were performed using R3.2.3. The prevalence of MetS was estimated in the population by age decade and follow-up duration from HCT using two different definitions (IDF and ATP III) [1, 2]. Univariate comparison of patients and HCT characteristics between the two groups (MetS vs no MetS) was performed using the non-parametric Mann–Witney *U* test for continuous variables and Chi-square test or Fisher’s exact test for categorical variables. All tests were two-sided. Multivariate logistic regression analysis was performed to adjust for confounding factors that may have an impact on the association between MetS and previous CVE. Variables with a *p* value <0.2 in univariate analysis were included. The results are expressed as odds ratio (OR) with a 95% confidence interval (95% CI). The association of the two MetS definitions with the presence/absence of previous CVE was analysed using Receiver Operating Characteristic (ROC) curves and DeLong’s test to compare Area Under the Curves (AUC).

## Results

### Demographics

Four hundred and fifty-three patients (258 men and 195 women) were evaluated (366, 80.8% allogeneic and 87, 19.2% autologous HCT). Table 1 gives the population demographics in terms of age, gender, ethnicity, primary diagnosis, conditioning type, ECOG status at time of evaluation (i.e. baseline), and history of second malignancy. Two patients labelled as having plasma cell disorders were entered inadvertently by two centres and were identified after data cleansing. Their results were not outlying and thus, for pragmatic reasons, their data remained in the analysed dataset.

**Table 1:** Patient demographics.

Variable	Overall	Allogeneic	Autologous
	(*n* = 453)	(*n* = 366)	(*n* = 87)
Patient age at diagnosis (years), median (IQR 25–75)	40.6 (29.5–50.7)	41.2 (30.5–51.1)	37.6 (28.9–48.6)
Patient age at transplant (years), median (IQR 25–75)	43.3 (31.8–53.1)	44.1 (32.3–53.3)	39.3 (31.3–50.5)
Patient Age at follow up (years), median (IQR 25–75)	52.1 (40.7–60.6)	52.0 (40.5–60.5)	52.5 (41.6–61.3)
Survival (years), median (IQR 25–75)	6.14 (3.27–10.6)	5.7 (3.2–9.6)	9.7 (4.5–16.9)
Gender, *n* (%)			
Male	258 (57.0)	212 (57.9)	46 (52.9)
Female	195 (43.0)	154 (42.1)	41 (47.1)
Diagnosis, *n* (%)			
Acute leukaemia	172 (38.0)	162 (44.3)	10 (11.5)
Chronic leukaemia	57 (12.6)	57 (15.6)	0 (0.0)
Lymphoma	125 (27.6)	51 (13.9)	74 (85.1)
Plasma cell disorders	22 (4.9)	20 (5.5)	2 (2.3)
Solid tumours	1 (0.2)	0 (0.0)	1 (1.2)
Myelodysplastic/Myeloproliferative	58 (12.8)	58 (15.8)	0 (0.0)
Bone marrow failure	17 (3.8)	17 (4.6)	0 (0.0)
Inherited disorders	1 (0.2)	1 (0.3)	0 (0.0)
Ethnicity, *n* (%)			
European Caucasian	444 (98.7)	358 (98.6)	86 (98.9)
South and South-East Asian	4 (0.9)	3 (0.8)	1 (1.1)
Ethnic South and Central American origins	1 (0.2)	1 (0.3)	0 (0.0)
African Caribbean	1 (0.2)	1 (0.3)	0 (0.0)
Missing	3	3	0
ECOG at time of evaluation, *n* (%)			
0	333 (76.2)	267 (76.1)	66 (76.7)
1	90 (20.6)	74 (21.1)	16 (18.6)
2	11 (2.5)	8 (2.3)	3 (3.5)
3	2 (0.5)	1 (0.3)	1 (1.2)
4	1 (0.2)	1 (0.3)	0 (0.0)
Missing	16	15	1
Conditioning type (allo only), *n* (%)			
MAC		158 (47.6)	
RIC-Chemo		150 (45.2)	
RIC-TBI		24 (7.2)	
Missing		36	
Second malignancy (any kind), *n* (%)			
No	392 (89.7)	316 (89.0)	76 (92.7)
Yes	45 (10.3)	39 (11.0)	6 (7.3)
Missing	16	11	5

*IQR* interquartile range, *MAC* myeloablative conditioning, *RIC* reduced intensity conditioning, *TBI* total body irradiation.

### Relationship of MetS definition with CV events

To establish which definition of MetS is appropriate for use related to HCT survivors, we investigated the presence of criteria in a sub-cohort already identified as having a CVE [29] using ROC analysis to compare the AUC between the ability of ATPIII and IDF metabolic criteria for an “ever” CVE using DeLong’s test. Neither MetS definition was significantly associated with the occurrence of a previous CVE (*p* = 0.65).

### Prevalence of MetS

We present the prevalence of MetS using both definitions and then present further results related to the IDF definition only, since we found no superiority (using ROC analysis) of one definition over another and the given published preference for using the IDF definition [7].

Using both the harmonised (IDF) definition of MetS (at least 3/5 factors), and the ATPIII definition, the prevalence of MetS was 37.5% and 43.5% respectively. The prevalence rate for allogeneic HCT patients was 36.4% using IDF and 42.9% using ATPIII definitions, and for autologous HCT patients was 42.3% and 46.2% for each definition, respectively. Table 2 gives the prevalence rates of MetS by each definition and for each of the MetS risk factors for the whole cohort, and by transplant type.

**Table 2:** Prevalence of metabolic syndrome and its component factors.

Variable	Overall (*n* = 453)	Allogeneic (*n* = 366)	Autologous (*n* = 87)	*P*
Metabolic syndrome—IDF definition, *n* (%)				0.33
No	255 (62.5)	210 (63.6)	45 (57.7)	
Yes	153 (37.5)	120 (36.4)	33 (42.3)	
Missing	45	36	9	
Metabolic syndrome—ATPIII definition, *n* (%)				0.56
No	226 (56.5)	184 (57.1)	42 (53.9)	
Yes	174 (43.5)	138 (42.9)	36 (46.2)	
Missing	53	44	9	
Waist circumference, *n* (%)				0.55
Normal	149 (33.0)	123 (33.6)	26 (30.2)	
Elevated	303 (67.0)	243 (66.4)	60 (69.8)	
Missing	1	0	1	
Triglycerides, *n* (%)				0.30
Normal	186 (48.6)	140 (47.1)	46 (53.5)	
Elevated	197 (51.4)	157 (52.9)	40 (46.5)	
Missing	70	69	1	
HDL cholesterol, *n* (%)				0.50
Normal	273 (63.8)	220 (63.0)	53 (67.1)	
Low	155 (36.2)	129 (37.0)	26 (32.9)	
Missing	25	17	8	
Blood pressure, *n* (%)				0.30
Normal	176 (39.0)	147 (40.2)	29 (34.1)	
Elevated	275 (61.0)	219 (59.8)	56 (65.9)	
Missing	2	0	2	
Non-fasting glucose, *n* (%)				0.28
Normal	371 (88.8)	301 (89.6)	70 (85.4)	
Elevated	47 (11.2)	35 (10.4)	12 (14.6)	
Missing	35	30	5	

There was a significant difference in prevalence of MetS by age at diagnosis, age at HCT and age at follow-up (all *p* < 0.001 with increasing age) using both definitions. Logistic regression showed there was an influence of increasing age at follow up in the prevalence of MetS using the IDF definition (odds ratio (OR) 8.7, 95% CI 4.2,18.1) for the > 50 years age group compared with those aged 18–29 years. Statistically significant differences in BMI were observed between patients with and without MetS (28.2 [IQR 25.6–31] vs 23.3 [IQR 21.3–25.9], respectively, *p* < 0.001; results not shown).

### Relationship of MetS with history of CV disease

Notably, irrespective of the type of HCT, there was a significantly higher frequency of history of CVE (cerebrovascular accident, coronary heart disease and peripheral vascular disease) in those with MetS than in those without (26.7.vs 9.0%, *p* < 0.0001). Eighteen out of 210 (8.6%) allogeneic HCT patients without MetS reported at least one CVE compared with 30/120 (25%) of patients with MetS (*p* < 0.0001). Similarly, in autologous HCT patients, 4/45 (8.9%) without MetS reported a CVE as compared to 9/33 (27.3%) with MetS (*p* = 0.03). Logistic regression analysis showed an influence of increasing age at transplant with increasing prevalence of CVE (OR 3.49, 95% CI 1.54, 7.89, *p* = 0.003) for the over 50 s compared with those aged 18–29 years at transplant (results not shown).

### Relationship of MetS with pre-transplant risk factors for CV disease

There was no relationship between prevalence of MetS (following HCT) and reported family history of CV disease, or with reported alcohol use.

Smoking status was reported as current for 44 patients, 155 reported being previous smokers, and 205 as never smokers. For patients with MetS, the OR for smoking >10 pack years compared with smoking ≤10 pack years or less was 2.7 (95% CI 1.68, 4.33, *p* < 0.001).

Of 45 patients with pre-existing type II diabetes, 35 had MetS and 10 did not.

### Relationship of MetS with HCT intensity

For patients who received an allogeneic HCT, no relationship between prevalence of MetS and conditioning intensity was observed between the following groups: those who received myeloablative conditioning (*n* = 158) and those who received RIC (*n* = 174); those who received TBI (*n* = 129) and those who did not (*n* = 235); those who had myeloablative conditioning with chemotherapy only (*n* = 73) and those who had myeloablative conditioning with TBI (*n* = 85). The median age at treatment for those who received myeloablative conditioning was 37.7 years (IQR 27.6–46.7) compared with 51.1 for those who received RIC (IQR 42.1–57.5). Similarly the median age at treatment for those who received TBI was 38.1 years (IQR 29.5–46.5) compared with 46.1 years who did not receive TBI (IQR 33.1–55.1).

### Relationship of MetS with medication use

No association was evident between prevalence of MetS and current or previous use of immunosuppressant therapy (*p* = 0.23), significant corticosteroid treatment use (e.g. prednisolone, methylprednisolone, dexamethasone) for more than 28 days post HCT (*p* = 0.6) or hormone replacement use (i.e. oral contraceptive pill or hormone replacement in women, or testosterone replacement in men) (*p* = 0.87; results not shown).

### Relationship of MetS with GvHD

For patients who had received allogeneic HCT, no relationship was observed between prevalence of MetS and confirmation (answered as yes/no) of grade II to IV aGvHD (*p* = 0.82) or cGvHD (*p* = 0.66; results not shown).

### Second or newly occurring malignancies

Forty-five of 392 patients were reported to have a second or newly occurring malignancy recorded post HCT (answered as yes/no, without specification of tumour type). There was no observed difference between those with and without a second malignancy and the prevalence of MetS (*p* = 0.10). Unexpectedly, univariate logistic regression analysis showed that a CVE with occurrence of a second malignancy carried an OR of 2.83 (95% CI 1.38, 5.81 *p* = 0.005).

## Discussion

This cross-sectional, non-interventional survey represents the largest multi-centre ‘real life’ evaluation of MetS in HCT survivors and confirms a high prevalence of MetS following both allogeneic and autologous HCT; 37.5% overall rising to 52.8% in those aged over 50 years at follow-up. The data support MetS being an age-related late effect of HCT strongly associated with CVE.

This prevalence concurs with previous smaller single centre studies in adult HCT practice [31, 32] irrespective of type of HCT. Based on a previous study [18], a higher prevalence of MetS and CVE in allogeneic HCT patients compared with autologous HCT might have been anticipated given the generally more intensive nature of allogeneic HCT treatment, with a higher rate of subsequent complications such as GvHD and its treatments, which include corticosteroids, calcineurin inhibitors and other drugs with CV risk. However, our study did not support the prevalence of MetS being significantly related to these factors. Although there was an effect of increasing prevalence with increasing age demonstrated in the multivariate analysis (i.e. lower in the allogeneic HCT cohort) and/or patient selection (i.e. lower co-morbidities in the allogeneic HCT group), MetS and CV risk similarly affected both autologous and allogeneic HCT survivors. Therefore, consideration of MetS should extend beyond the ‘curative intent’ groups (NHL and HL) included in this study to other patients undergoing autologous HCT.

In our study ATP III and IDF definitions were not significantly different or in their association with a CVE, consistent with other reports in the general population. IDF defines MetS features *below treatment thresholds*, therefore allows earlier identification of ‘at-risk’ patients [33]. We chose the IDF definition because in terms of performance using the ROC analysis, not one definition was superior to the other meaning we defaulted to the IDF, the international definition of choice [7].

The association between the occurrence of second malignancy and CV disease is notable. Although we did not find an association between MetS and cancer as such, MetS has been associated with many cancers including breast, pancreatic, colon and liver, and each individual risk factor for MetS has also an association with cancer [7]. Alternatively, time after transplant may be a confounding variable, reflecting an increase in both malignancies and in CV disease in the years after HCT. Future studies to elucidate the association of MetS and second malignancy are required, including specific tumour types, as this information may be useful for long-term lifestyle advice post-HCT.

Our study is limited in a number of respects, particularly by its cross-sectional nature. We were not able to establish the time of onset of MetS in relation to either a pre-existing prevalence or the treatment/transplant received. It is likely with a longer follow-up time and longitudinal sequential evaluation, the greater the probability of CVE. Caution is required in comparisons between allogeneic and autologous HCT because of different indications and also regarding risk of second malignancy since all cancer types, including skin cancers were included in the analysis. There were also procedural limitations, including the non-interventional evaluation of patients in ‘routine’ clinics. As such, we used the non-fasting cut-offs for blood glucose defined by the American Diabetes Association guidance [30], which may have underestimated levels of diabetes. Furthermore, registry data might not have been fully complete (for example accuracy of recording all secondary malignancies) and potentially affected by recall bias. There was also a possibility of recruitment bias linked to a motivated population attending follow-up/late effects clinic. In addition, two patients labelled as having plasma cell disorders were entered inadvertently from two centres and were identified after data cleansing. Their results were not outlying and thus, for pragmatic reasons, their data remained in the analysed dataset.

A further limitation to this study was a limitation in diversity of ethnic groups. Although differences in MetS by race and ethnicity are recognised, much of the research on management and prevention of MetS has focused almost entirely on European-derived populations and also in low and middle income countries [34]. Further studies should elucidate these aspects and implications for practice. Finally, the study lacked a control population.

Despite the limitations, this is the largest study of MetS in the setting of HCT and has implications for clinical and research practice. Although variably defined, MetS is a basis for on-going management of CV risk with lifestyle modification and pharmacological intervention. Screening and management of MetS and CV risk should be integrated within models of routine long-term follow-up care in both allogeneic and autologous HCT according to international guidelines and recommendations [28, 35].

Early intervention of reversible features of MetS with lifestyle and medical management may reduce CVE, but this needs to be tested prospectively, and ultimately in randomised controlled trials in HCT patients. Scoring systems for CV disease, like Framingham [36] and QRISK [37], used commonly in the general population, require validation in the HCT population, and ‘bespoke’ systems need to be developed.

In conclusion, our study has confirmed a high prevalence of MetS in both allogeneic and autologous HCT survivors, and a strong association between MetS and CVE. Further clinical and scientific research is warranted into patient, donor, transplant technique, graft source, pre-treatment, immunosuppression or other potential influencing factors, as well as mechanisms. In the meantime, screening should be incorporated in long-term follow up of all HCT patients.
